# MCP-1 Levels are Associated with Cardiac Remodeling but not with Resistant Hypertension

**DOI:** 10.5935/abc.20170033

**Published:** 2017-04

**Authors:** Alessandra Mileni Versuti Ritter, Ana Paula Cabral de Faria, Andrea Sabbatini, Nathalia Batista Corrêa, Veridiana Brunelli, Rodrigo Modolo, Heitor Moreno

**Affiliations:** Universidade Estadual de Campinas (UNICAMP), Campinas, SP - Brazil

**Keywords:** Refractory Hypertension, Cytokines, Monocyte Chemoattractant Proteins, Left Ventricular Hypertrophy

## Abstract

**Background:**

Hypertension is a chronic, low-grade inflammation process associated with the release of cytokines and development of target organ damage. Deregulated monocyte chemoattractant protein-1 (MCP-1) levels have been associated with high blood pressure and cardiovascular complications; however, the mechanisms involved are complex and not fully understood.

**Objective:**

This study aimed to compare the levels of MCP-1 in patients with resistant (RH) versus mild-to-moderate (HTN) hypertension and their association with the presence or absence of left ventricular hypertrophy (LVH) in all hypertensive subjects.

**Methods:**

We enrolled 256 hypertensive subjects: 120 RH and 136 HTN, investigating the relationship between circulating MCP-1 levels and blood pressure, biochemical data, hematologic profile, and cardiac damage within the RH and HTN groups. Plasma MCP-1 levels were measured by ELISA and LVH was assessed by echocardiography.

**Results:**

We found no difference in MCP-1 levels between RH and HTN subjects. On the other hand, we encountered lower MCP-1 levels in patients with LVH (105 pg/mL [100 - 260 pg/mL] versus 136 pg/mL (100 - 200 pg/mL), p = 0.005, respectively] compared with those without LVH. A logistic regression model adjusted for body mass index (BMI), age, race, aldosterone levels, and presence of diabetes and RH demonstrated that median levels of MCP-1 (2.55 pg/mL [1.22 - 5.2 pg/mL], p = 0.01) were independently associated with LVH in the entire hypertensive population.

**Conclusion:**

Since MCP-1 levels were similar in both RH and HTN subjects and decreased in hypertensive patients with existing LVH, our study suggests a possible downregulation in MCP-1 levels in hypertensive individuals with LVH, regardless of hypertension strata.

## Introduction

Resistant hypertension (RH) is defined as a condition in which patients present either (i) uncontrolled blood pressure (BP) (≥ 140/90 mmHg) despite the use of maximal recommended or tolerated doses of three or more antihypertensive drugs, or (ii) controlled BP with the use of at least four medications.^1,2^ The high prevalence of target organ damage (TOD), such as left ventricular hypertrophy (LVH), is higher in patients with RH compared with those with mild/moderate hypertension (HTN)^1,3^ and is considered a predictor of future cardiovascular events in this specific RH population.^4^

Many lines of evidence have established that hypertension is a chronic low-grade inflammation process that plays a role in the development and maintenance of TOD.^5,6^ Several inflammatory mediators are enhanced in hypertensive subjects,^7^ including monocyte chemoattractant protein-1 (MCP-1).^8^ MCP-1, also known as CCL2, can be produced by different cells and is responsible for migration of monocytes and macrophages cells to the tissue,^9^ exacerbating the local damage.

Experimental models of hypertension have shown that infiltration of inflammatory cells (macrophages) in the vascular walls is strongly related to increased BP^10^ and cardiovascular alterations.^11,12^ A clinical study has suggested that MCP-1 levels may vary according to the degree of hypertension,^8^ indicating a stage-dependent biomarker of the disease.

Although some authors have shown that increased levels of MCP-1/CCL2 and macrophages in the heart contribute to cardiac damage,^13,14^ others have pointed out that macrophages have cardioprotective effects.^15^ In fact, one study showed that depletion of macrophages accelerates the development of cardiomyopathy in hypertensive rats.^15^ This effect could be explained by an ability to maintain cardiac homeostasis during some cardiac diseases.^14^

Despite these findings, the relationship of MCP-1 with RH and cardiac damage in the clinical setting has not been established yet. Therefore, this study was designed to assess the levels of MCP-1 in RH compared with HTN subjects and its association with LVH in all hypertensive groups.

## Methods

### Study subjects

A convenience sample of 256 hypertensive patients from the Resistant Hypertension Outpatient Clinic at University of Campinas (Campinas, Brazil) were enrolled in this cross-sectional study.

Patients were diagnosed with RH after a 6-month protocol to exclude pseudoresistance - white-coat hypertension and poor medication adherence - with ambulatory BP monitoring (ABPM), the Morisky questionnaire, and pill count. Secondary hypertension (renal artery stenosis, pheochromocytoma, and primary hyperaldosteronism) was also excluded. These subjects were enrolled in the RH group. Also, patients with controlled BP using three or less antihypertensive drugs, or not yet controlled using two or less of these medications were classified as having HTN and also enrolled in the study.

 The patients were classified into two groups: RH (n = 120) and HTN (n = 136). In addition, we combined both RH and HTN groups together and assessed the MCP-1 levels according to (1) the presence or absence of LVH (115 g/m^2^ for men and 95 g/m^2^ for women)^16^ and (2) LVH severity, considering patients without LVH as level 0; patients with LVH and left ventricular (LV) mass index (LVMI) < median (121 g/m^2^) as level 1; and patients with LVH and LVMI ≥ median (121 g/m^2^) as level 2.

All ethical requirements for experiments conducted in human subjects were strictly followed. The study was approved by the Research Ethics Committee at Faculty of Medical Sciences, University of Campinas (São Paulo, Brazil) (approval no. 1.112.881/2015) and was conducted in accordance with the Declaration of Helsinki. All participants signed a written informed consent form before study enrollment.

### Office blood pressure measurements

A trained health professional measured the office BP at least three times using a certified digital sphygmomanometer (HEM-907 XL OMRON Healthcare Inc., Bannockburn, IL, USA) in accordance with the 2013 European Society of Hypertension (ESH) guidelines.^17^ The average of two or three consecutive measurements was used if a difference between the measurements was below 5 mmHg.

### Ambulatory blood pressure monitoring

24-hour ABPM was carried out using an automatic oscillometric device (Spacelabs 90207, Spacelabs Inc.). The measurements were obtained every 20 minutes throughout the 24 hour period. The subjects were instructed to maintain their normal daily activities, avoid excessive physical activity, and take note of their sleep period in a personal diary. The mean BP was calculated during waking and sleep.

### Echocardiography

Experienced specialists blinded to the patients' clinical data measured echocardiographic parameters (Siemens Acuson CV70, Munich, Bavaria, Germany) using two-dimensional targeted M-mode echocardiography. Diastolic and systolic LV diameters and the interventricular septal and posterior wall thicknesses were measured according to the QRS wave of the electrocardiogram. The LV mass was calculated by the American Society of Echocardiography (ASE) recommended formula^18^ and the LVMI was calculated by dividing the LV mass by the body surface. An LVMI greater than 115 g/m^2^ for men and 95 g/m^2^ for women characterized the presence of LVH.^16^

### Serum collection and laboratory assessments

Blood samples were withdrawn from the antecubital vein, with atraumatic venipuncture, after 8 hours of overnight fasting. Plasma levels of MCP-1 were measured using enzyme-linked immunosorbent assay (ELISA; R&D Systems, Inc., Minneapolis, MN, USA), according to the manufacturer's instructions. Radioimmunoassay (Immunotech SAS, Marseille, France) was used to measure the plasma level of aldosterone according to the manufacturer's instructions. The neutrophil/lymphocyte ratio (NLR) was calculated by absolute neutrophil count divided by absolute lymphocyte count. In addition, serum total cholesterol, low- and high-density lipoprotein cholesterol, triglycerides, glucose, and creatinine were measured. Creatinine clearance (mL/min/1.73 m^2^) was measured in a urine sample collected during 24 hours.

### Statistical analysis

Descriptive data are shown as mean ± standard deviation (SD) for parametric data or median (interquartile range [IQR]) for nonparametric data. The distribution of the data was assessed by the Shapiro-Wilk test. Non-paired Student's *t* test or Mann-Whitney test was performed to compare two groups, while Kruskal-Wallis or analysis of variance (ANOVA) test, followed by Dunn's or Bonferroni post-test, respectively, were used for groups of three, according to data distribution. Categorical variables are presented in frequencies and/or percentages and were compared by Fisher's test. Spearman's correlation tested the association of nonparametric data. Also, we performed multiple logistic regression for the presence of LVH adjusted for age, aldosterone levels, body mass index (BMI), race, presence of diabetes, presence of RH, and MCP-1 median levels (categorized according to the median value of < 125 pg/mL) in hypertensive subjects. The level of statistical significance taken into account was < 0.05.

The analyses were performed using the software SigmaPlot (Systat Software, Inc, v.12, Chicago, IL, USA).

## Results

Table 1 shows the general characteristics, biochemical data, and hematologic profile of the 256 hypertensive subjects. We found a higher percentage of diabetic individuals and black race in the RH compared with the HTN group. Moreover, RH patients showed higher office systolic BP (SBP) and aldosterone levels, a higher incidence of LVH, and imbalance of lipid and glucose profiles compared with HTN subjects. On the other hand, we found no difference in hematologic parameters between the groups.

**Table 1:** General characteristics of the subjects with resistant and mild-to-moderate hypertension

Hypertensive (n = 256)			
	HTN (n = 136)	RH (n = 120)	p value
**Clinical Data**			
Age (years)	65 ± 10	60 ± 11	< 0.001
Women (%)	62	67	0.50
Black race (%)	13	44	< 0.001
Diabetes (%)	38	51	0.05
Office SBP (mmHg)	139 (131 – 148)	147 (134 – 160)	< 0.001
Office DBP (mmHg)	81 (76 – 86)	83 (78 – 92)	0.09
ABPM SBP (mmHg)	127 (118 – 135)	130 (117 – 143)	0.18
ABPM DBP (mmHg)	76 (70 – 81)	77 (70 – 83)	0.34
LVMI (g/m^2^)	100 (87 – 119)	113 (95 – 142)	< 0.001
**Biochemical Data**			
C-reactive protein (mg/dL)	0.3 (0.2 – 0.6)	0.3 (0.2 – 0.6)	0.72
Cholesterol (mg/dL)	165 (140 – 185)	181 (151 – 209)	0.003
HDL (mg/dL)	49 (42 – 57)	46 (38 – 54)	0.02
LDL (mg/dL)	87 (67 – 107)	98 (79 – 127)	0.002
Triglycerides (mg/dL)	108 (80 – 151)	129 (93 – 185)	0.019
HbA1c (%)	6.0 (5.8 – 6.5)	6.3 (6.0 – 7.8)	0.007
Glucose (mg/dL)	97 (90 – 107)	101 (89 – 132)	0.12
Creatinine (mg/dL)	0.94 (0.8 – 1.1)	0.97 (0.8 – 1.2)	0.15
Creatinine clearance (mL/min/1.73m^2^)	65 (28 – 93)	81 (62 – 98)	0.05
Aldosterone (ng/dL)	68 (43 – 115)	98 (60 – 179)	< 0.001
**Hematologic Profile**			
Leukocytes (mm^3^)	6.6 (6 – 8)	7.4 (6 – 8)	0.03
Monocytes %	8 (7 – 9)	8 (6 – 9)	0.79
Lymphocytes %	30 ± 7	30 ± 8	0.85
Basophils %	0.4 (0.2 – 0.5)	0.4 (0.3 – 0.6)	0.41
Eosinophils %	3 (2 – 4)	2 (1 – 3)	0.43
Neutrophils %	59 ± 7	58 ± 10	0.60
NLR	2 (1.8 – 2.3)	2 (1.4 – 2.6)	0.80

HTN: mild–to-moderate hypertensive subjects; RH: resistant hypertensive subjects; SBP: systolic blood pressure; DBP: diastolic blood pressure; ABPM: ambulatory blood pressure measurement; LVMI: left ventricular mass index; HDL: high-density lipoprotein; LDL: low-density lipoprotein; HbA1c: glycated hemoglobin; NLR: neutrophil/lymphocyte ratio; Continuous variables are presented as mean ± standard deviation (SD) for parametric data or median (1st, 3rd quartiles) for nonparametric data. Categorical variables are presented as percentages. Student’s t test or Mann Whitney test was performed according to data distribution, and Fisher’s test was used to compare categorical variables

We observed that RH individuals used a greater number of antihypertensive drugs and a higher proportion of almost all antihypertensive classes, except for angiotensin II receptor antagonists (ARAs) (Table 2). Furthermore, the number of individuals using statins was greater in the HTN compared with the RH group.

**Table 2:** Medication use in resistant and mild-to-moderate hypertensive subjects

Hypertensive (n = 256)			
	HTN (n = 136)	RH (n = 120)	p value
**Antihypertensive drugs**			
Number of classes	2 (2 – 3)	4 (4 – 5)	< 0.001
Diuretics (%)	66	91	< 0.001
Spironolactone (%)	2	40	< 0.001
ACEIs (%)	16	37	< 0.001
ARBs (%)	74	55	0.003
CCBs (%)	46	84	< 0.001
Beta-blockers (%)	14	69	< 0.001
**Others drugs**			
Hypoglycemic agents (%)	38	51	0.05
Statin (%)	75	57	0.003

HTN: mild–to-moderate hypertensive subjects; RH: resistant hypertensive subjects; ACEIs: angiotensin-converting enzyme inhibitors; ARBs: angiotensin receptor blockers; CCBs: calcium channel blockers. Categorical variables are presented as numbers and percentages. Fisher’s test was performed to compare categorical variables.

**Figure 1:**
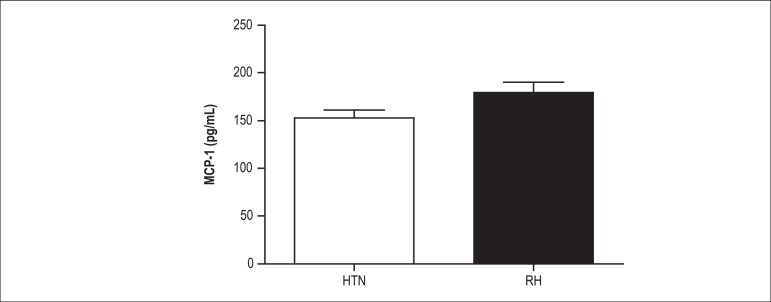
Plasma MCP-1 levels in subjects with resistant hypertension (RH, n = 119, 153 ± 93 pg/mL) and mild-to-moderate hypertension (HTN, n=114, 178 ± 120 pg/mL, p = 0.47). Values are expressed as mean ± standard deviation (SD).

**Figure 2:**
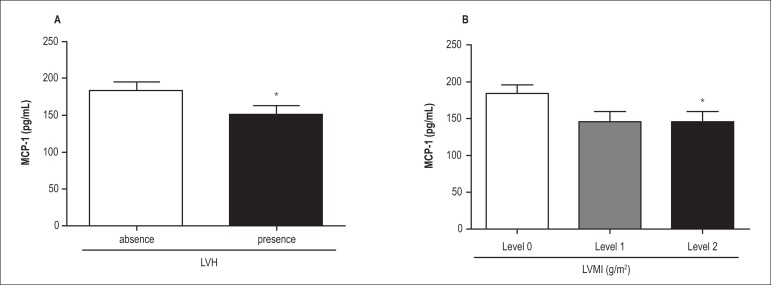
Plasma MCP-1 levels according to (A) presence (n = 96) or absence (n = 94) of left ventricular hypertrophy (LVH; cut-off value of 115 g/m^2^ for men and 95 g/m^2^ for women) and (B) level of LVH in all hypertensive subjects (with resistant hypertension and mild-to-moderate hypertension): level 0 = patients without LVH (left ventricular mass index [LVMI] < 115 g/m^2^ in men and < 95 g/m^2^ in women); level 1 = patients with LVH and LVMI levels < 121 g/m^2^; and level 2 = patients with LVMI levels ≥ 121 g/m^2^. Values are expressed as mean ± standard deviation (SD). (A) *p = 0.005 compared with the absence of LVH and (B) *p = 0.01 compared with level 0.

Regarding MCP-1 levels, we found no differences between RH and HTN subjects (153 ± 93 pg/mL *versus* 178 ± 120 pg/mL, respectively, p = 0.47) (Figure 1). However, when we combined both RH and HTN groups together and assessed the MCP-1 levels according to the presence or absence of LVH, we found lower MCP-1 levels in patients with LVH compared with those without LVH (105 pg/mL [100 - 260 pg/mL] *versus* 136 pg/mL [100 - 200 pg/mL], respectively, p = 0.005) (Figure 2A). Also, when we stratified the LVMI levels into three degrees of LVH severity, we found that patients with the highest degree of hypertrophy (LVMI > 125 g/m^2^ - level 2) showed lower MCP-1 levels compared with those with the lowest degree (levels 0 and 1) (Figure 2B). Also, the subjects at the lowest (level 0) and intermediate levels (level 1) of LVH demonstrated similar MCP-1 levels.

Finally, the logistic regression model demonstrated that MCP-1 levels were inversely associated with the presence of LVH after adjustment for BMI, age, race, aldosterone level, and the presence of diabetes and RH (Table 3).

**Table 3:** Multiple logistic regression for the presence of left ventricular hypertrophy (LVH) in all hypertensive subjects (resistant hypertension and mild-to-moderate hypertension)

Variable	Odds ratio (95% confidence interval)	p value
MCP-1 < 125 pg/mL	2.55 (1.2 – 5.2)	0.01
Presence of diabetes	0.64 (0.3 – 1.1)	0.21
Resistant hypertension	3.7 (1.5 – 8.6)	0.003
Aldosterone (ng/dL)	1.0 (1 – 1)	0.97

MCP-1 was categorized by median levels.

## Discussion

The main finding of this study was the association between MCP-1 levels and presence of LVH in hypertensive subjects, especially in those with advanced LVH, regardless of resistance to antihypertensive treatment.

Strong evidence supports the role of the inflammatory process in hypertension.^19^ Both RH and HTN patients present higher levels of inflammatory cytokines and their levels are related to TOD.^6^ MCP-1 is a proinflammatory cytokine with potent chemoattractant activity for monocytes and macrophages.^5^ The recruitment and activation of monocytes in rat models appear to be involved with hypertension and TOD process^20^ by increasing oxidative stress in the vascular wall.^10^ Moreover, mice lacking MCP-1 receptor present no cardiac fibrosis or fibroblast accumulation in the heart after angiotensin infusion,^21^ suggesting that MCP-1 and its receptor may have an important role in cardiac damage.

A recent study demonstrated that RH individuals have higher levels of MCP-1 compared with normotensive subjects.^22^ However, we found no differences in MCP-1 levels between RH and HTN subjects, suggesting no association between MCP-1 and resistance to antihypertensive drugs.

Regarding MCP-1 levels in hypertensive patients, the data are limited and conflicting.^11,23^ One study has shown higher levels of MCP-1 in patients with untreated hypertension compared with controls and patients with isolated systolic hypertension.^23^ Mirhafez et al.^8^ proposed that cytokines are dependent on the hypertension stage. Indeed, these authors found similar MCP-1 levels among normotensive, pre-hypertensive, and stage 2 hypertensive subjects, but higher MCP-1 levels in stage 1 hypertensive ones compared with their controls.^24^ On the other hand, we found similar MCP-1 levels between RH and HTN subjects despite SBP differences between the groups. Also, a multiple logistic regression analysis showed no influence of BP levels in circulating MCP-1 after adjustment for potential confounders (data not shown).

It is well described that RH subjects represent a group with unfavorable phenotype compared with HTN ones. Therefore, the RH subgroup was expected to have high aldosterone levels,^1^ presence of LVH,^25^ and a greater number of individuals of black race, since these are characteristics closely related to the presence of RH. However, there are no data in the literature showing influence of these parameters on MCP-1 levels.

Equally to the similar MCP-1 levels in our groups, we found no difference in C-reactive protein levels and the number of monocytes between RH and HTN subjects, showing that the inflammatory state may be similar in both groups, corroborating other studies that found no difference in some inflammatory mediators between these groups.^6,26,28^ The similar findings between RH and HTN may indicate a BP-independent inflammatory process.

Cardiac damage is an adaptive response to chronic BP overload resulting in hypertrophic growth of cardiomyocytes.^29^ To date, the underlying mechanism involved in this TOD remains unknown, although evidence supports the fact that specialized inflammation cells - including monocytes - contribute to tissue lesion through cell-cell interaction performed by chemokines such as MCP-1.^30^

Recently, the idea that the innate immune system plays an important role in the initial and chronic phases of cardiac injury has been brought to knowledge. An experimental study in the early inflammatory phase of infarct healing has shown a marked upregulation of MCP-1 levels resulting in intense monocyte infiltration into the myocardium, while an opposite situation was observed in the chronic phase - a downregulation of MCP-1.^31^

Additionally, Weinberger et al.^12^ have shown that macrophages in the myocardium undergo dynamic changes in the course of life and the CCL-2 - receptor for MCP-1 - helps especially to identify macrophages that recently migrated from the circulation. Taken both studies together, we speculate that MCP-1 might also vary during TOD development in hypertension, where MCP-1 is downregulated in patients with LVH and with long-term hypertension. This may contribute to support our findings that MCP-1 may be differently regulated according to the degree of organ damage.

It is important to highlight that antihypertensive drugs have some antiinflammatory properties and may exert influence on chemokines.^32^ Consistent with these reports, a decrease in MCP-1 levels after administration of angiotensin-converting enzyme inhibitors (ACEIs)^33^ has been encountered. On the other hand, the use of losartan did not change MCP-1 levels.^33^ The authors suggested that only ACEIs could shift the MCP-1 levels by increasing oxide nitric and prostaglandin synthesis. [33] However, the precise mechanism still deserves further investigation.

Since RH subjects took a greater number of antihypertensive drugs compared with HTN ones, we assessed the potential influence of these medications on MCP-1 levels. A multiple linear regression analysis, adjusted for age, presence of LVH, and RH, revealed that only beta-blockers were independently associated with MCP-1 levels (beta coefficient = 55, standard error [SE] = 20, p < 0.01). Nevertheless, this possible interference might not have affected the outcome of our study, since RH subjects had similar MCP-1 levels as HTN ones, despite the use of a greater proportion of beta-blocker agents.

Since MCP-1 levels do not necessarily reflect their tissue concentration, this would be appointed as the main limitation of our study. We may also cite as limitations the lack of a normotensive control group and the possible interference of antihypertensive drugs in MCP-1 levels. However, due to ethical reasons, washout of these drugs in RH patients cannot be performed. Hence, as this is an observational study, we cannot infer a causal relationship between progression of the cardiac remodeling and changes in MCP-1 levels.

## Conclusion

The similar levels of cytokine betwen RH and HTN subjects and the lower MCP-1 levels in LVH patients suggest (i) a possible downregulation of MCP-1 levels in hypertensive patients with advanced stage of cardiac damage, and (ii) high activation of monocyte migration by MCP-1 in hypertensive patients developing cardiac structural changes. The modulation of chemokines represents an interesting therapeutic approach; therefore, further large clinical studies are required to define the potential involvement of the courses of hypertension and cardiac remodeling and changes in MCP-1 levels.
